# A Manual of Elementary Chemistry, Theoretical and Practical

**Published:** 1845-01

**Authors:** 


Art. IX.—*A Manual of Elementary Chemistry, Theoretical and Practical.* By George Fownes, ph. d., Chemical Lecturer in the Middlesex Hospital Medical School, and to the Pharmaceutical Society of Great Britain.—*London*, 1844. Flscp. 8vo, pp. 566.

This is another of the beautiful series of Manuals, for which the profession is indebted to our spirited publisher; and it is well worthy to take its place in the series. Having examined it with some attention, we feel qualified to recommend it to our younger readers as an admirable exposition of the present state of chemical science, simply and clearly written, and displaying a thorough practical knowledge of its details, as well as a profound acquaintance with its principles. The chief fault which we have to find with it,—in common with every chemical work that we have ever opened,—is, that the mass of details, of no interest or importance but to the professed chemist, is so great, as to weary the attention of the student, and to prevent him from thoroughly mastering that which it is essential for him to acquire. This is especially important in regard to medical students; the necessary demands upon whose time and mental application are so great, that as little as possible of either should be wasted upon what is superfluous. We could wish that Mr. Fownes had omitted some of these useless details, for the sake of extending the portions most useful to the medical student and practitioner,—those, namely, which explain the various processes of the Pharmacopœia, and which relate to the operations of animal and vegetable nutrition. All that he has written on these subjects is so good, that we cannot but desire to see it extended; even if the description of some hundred or two of compounds of which the student will never hear again, should be omitted to make room for it. The author has desired to give a practical character to the work, by giving a full and clear account of numerous laboratory processes. We could wish that he had added to his chapter on *ultimate* analysis (to which no medical *practitioner* can ever hope to give much attention) a similar code of directions for *proximate* analysis, a knowledge of which is becoming every day more important, and in which no one need despair of attaining tolerable success.

The illustrations, and the whole getting-up of the book, are fully equal to those of the precediug volumes of the series; than which we can give no higher praise.

